# Cleidocranial dysplasia presenting with retained deciduous teeth in a 15-year-old girl: a case report

**DOI:** 10.1186/1752-1947-6-25

**Published:** 2012-01-19

**Authors:** Nagarathna C, Bethur Siddaiah Shakuntala, Somy Mathew, Navin Hadadi Krishnamurthy, Ratna Yumkham

**Affiliations:** 1 Pedodontics and Preventive Dentistry, Rajarajeswari Dental College and Hospital, Bangalore- 560074, Karnataka, India

## Abstract

**Introduction:**

Cleidocranial dysplasia is a rare congenital defect of autosomal dominant inheritance caused by mutations in the *Cbfa1 *gene, also called *Runx2*, located on the short arm of chromosome 6. It primarily affects bones which undergo intramembranous ossification. This condition is of clinical significance to dentistry due to the involvement of the facial bones, altered eruption patterns and multiple supernumerary teeth.

**Case presentation:**

Our patient, a 15-year-old Indian girl, presented with the typical features of prolonged retention of deciduous dentition and delayed eruption of permanent teeth, that is, mandibular prognathism along with other skeletal abnormalities like shrugged shoulder and the absence of clavicles. A multidisciplinary approach was followed, comprising orthodontic, surgical and pedodontic teams for management.

**Conclusion:**

Successful treatment of such a case lies in a holistic approach that takes care of all aspects, including the primary pathology, the deformity itself and even the psychological angle.

## Introduction

Cleidocranial dysplasia is a rare congenital defect of autosomal dominance inheritance [1-3] that primarily affects bones which undergo intramembranous ossification. It was first described by Marie and Sainton in 1898 [4]. Cleidocranial dysplasia, also known as Marie and Sainton disease [5] or cleidocranial dysostosis [1], is associated with a spontaneous mutation in the gene encoding for transcription factor core binding factor alpha 1 (Cbfa1), which is essential for osteoblast and odontoblast differentiation as well as for bone and tooth formation [6]. The gene has been mapped to chromosome 6p21 [7].

The pathology relating to this condition is due to an early developmental disorder of mesenchyme or connective tissue. This causes retarded ossification of bone precursors, especially at junctions, which can lead to defective ossification, or even failure of ossification, of portions of the skeletal structure.

Cleidocranial dysplasia presents with skeletal defects, partial or complete absence of the clavicles, late closure of fontanelles, presence of open skull sutures and multiple wormian bones [2,8]. The maxilla is also underdeveloped along with ill-formed paranasal sinuses. This condition is of clinical significance to dentistry due to the involvement of the facial bones, altered eruption patterns and multiple supernumerary teeth. It manifests itself as a condition in which teeth fail to erupt, which is thought to be due to the absence of cellular cementum and an increase in the amount of acellular cementum of the roots of the affected teeth [9].

## Case presentation

**Figure 1:**
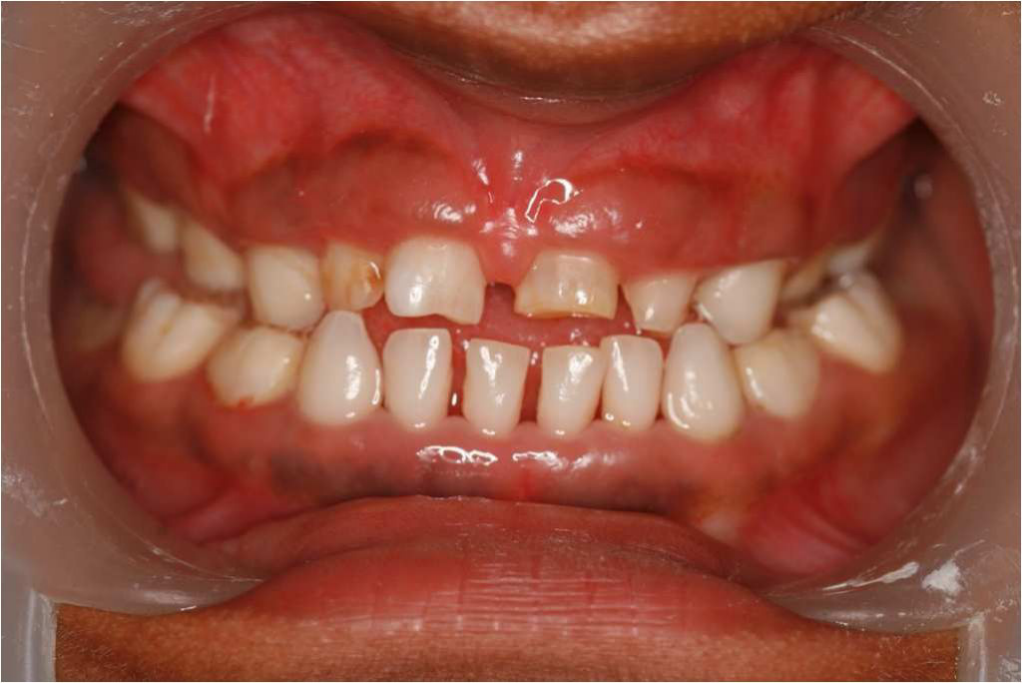
**Retained deciduous teeth**.

A 15-year-old Indian girl was referred to our Department of Pedodontics and Preventive Dentistry with the chief complaint of unerupted teeth. Her medical history revealed delayed closure of the anterior fontanelle, a fracture of her right humerus at three years of age and delayed puberty. Our patient was poorly built, short statured, moderately-nourished with a concave facial profile. She had shrugged shoulders with more than normal mobility of the shoulder girdle. Oral findings include Class III malocclusion with anterior and posterior crossbite and retained deciduous teeth (Figure 1).

**Figure 2:**
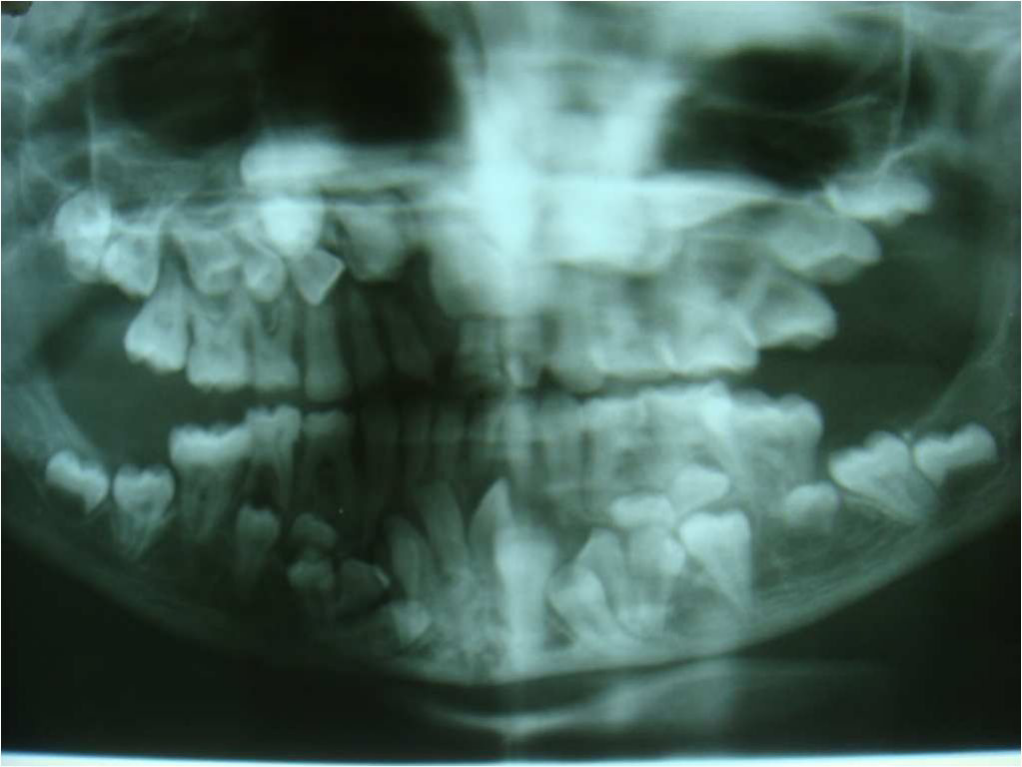
**Orthopantomogram showing impacted supernumeraries**.

**Figure 3:**
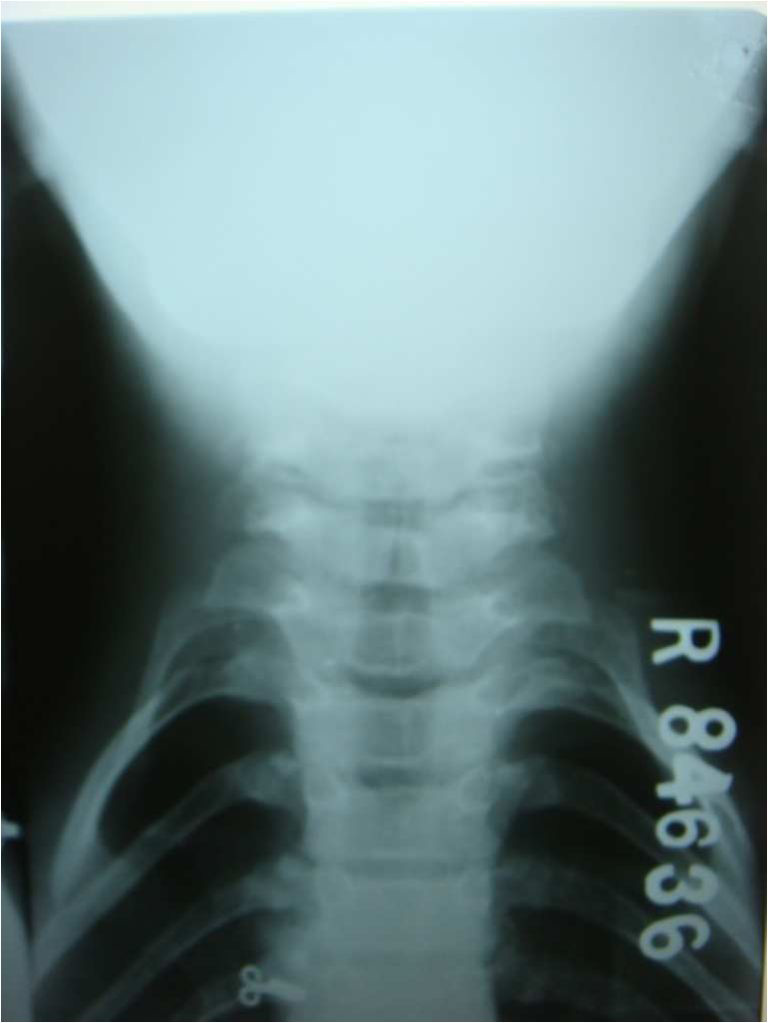
**Absence of the clavicle - one of the confirmatory feature of central core disease**.

**Figure 4:**
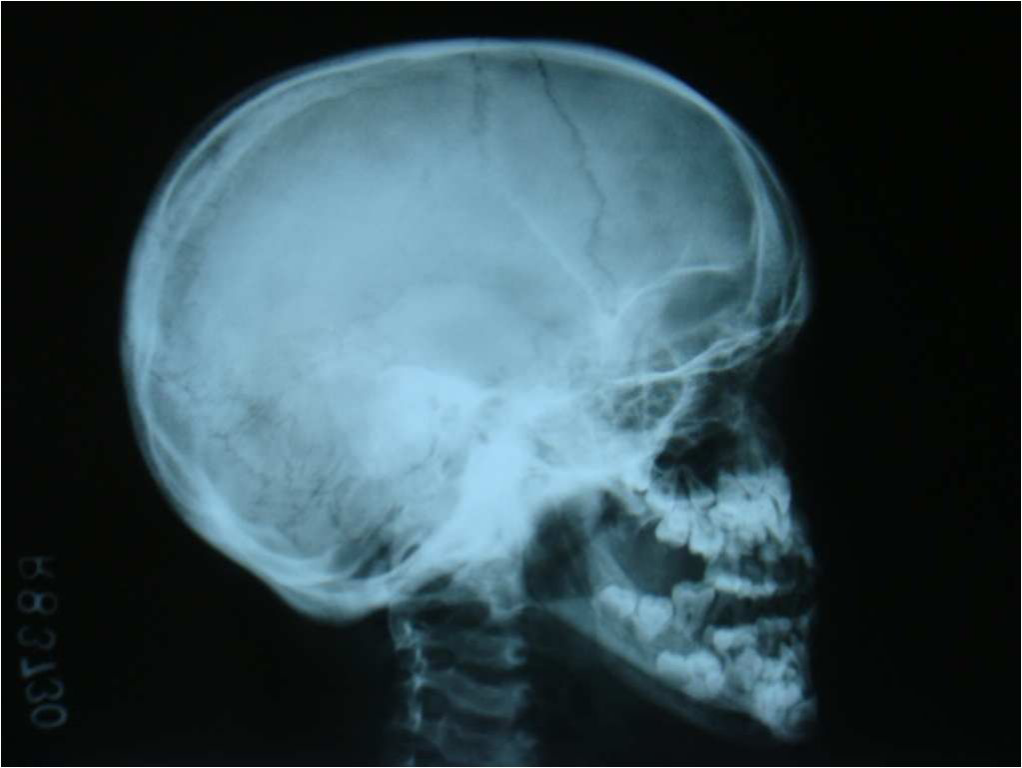
**Lateral cephalogram showing open skull sutures**.

An orthopantomogram revealed multiple unerupted permanent teeth and supernumerary teeth in the mandibular anterior region (Figure 2). A lateral cephalograph revealed wide skull sutures (Figure 3). The posteroanterior view of a chest radiograph revealed the absence of clavicles (Figure 4) and a bell-shaped ribcage. Based on these clinical and radiographic findings, a diagnosis of cleidocranial dysplasia was made. However, her chromosomal analysis revealed normal female karyotype 46XX.

**Figure 5:**
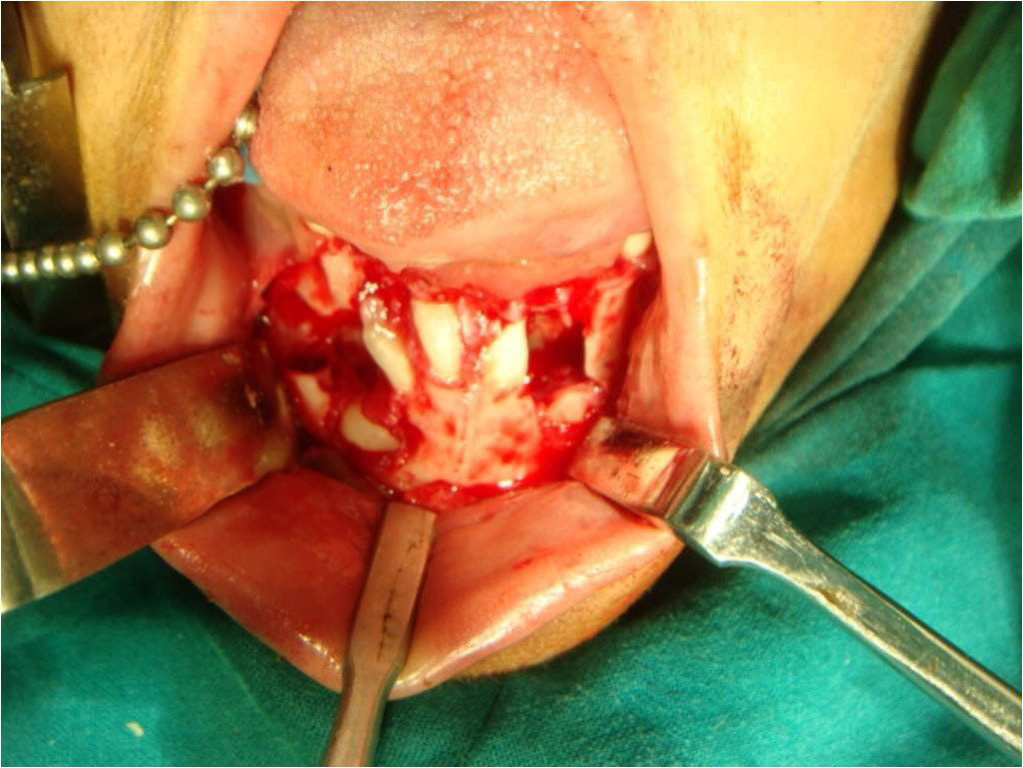
**Surgical removal of the retained mandibular deciduous teeth**.

**Figure 6:**
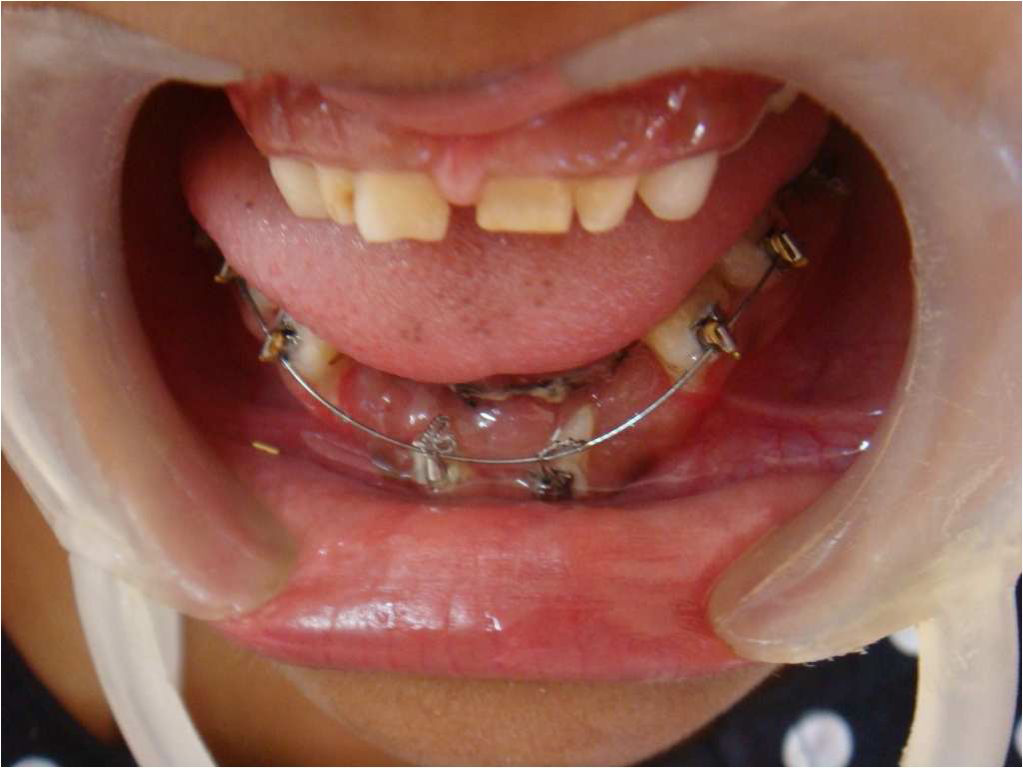
**Bonded brackets for orthodontic traction of permanent teeth to erupt**.

**Figure 7:**
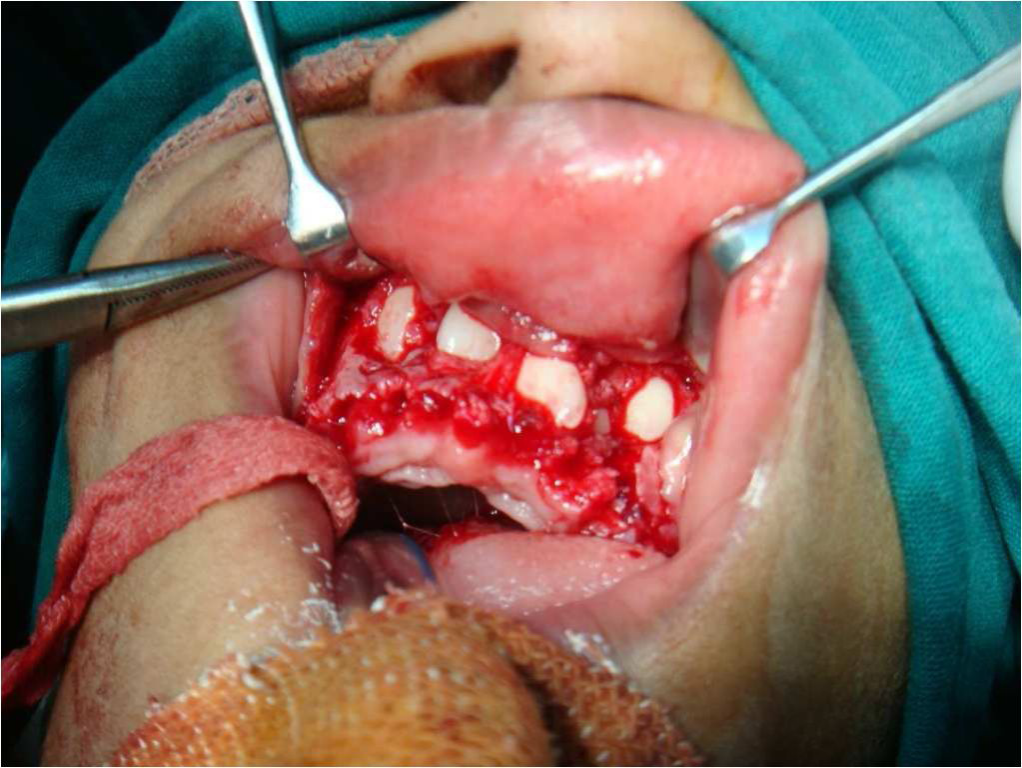
**Surgical removal of the retained maxillary deciduous teeth**.

A multidisciplinary dental approach involving oral and maxillofacial surgeons, orthodontists and pedodontists was followed in our case. Space management and proper eruption of her permanent teeth for aesthetic purposes were planned. Under general anesthesia, all her primary mandibular anterior teeth and supernumerary teeth were removed. Permanent anterior teeth were exposed surgically (Figure 5) and orthodontic brackets and ligature wires were placed for traction for the permanent teeth to erupt, along with a lingual arch appliance to prevent the arch collapsing (Figure 6). The same procedure was performed in the maxillary anterior region after two months (Figure 7). Our patient's self image was taken care of through behavior management and counseling.

**Figure 8:**
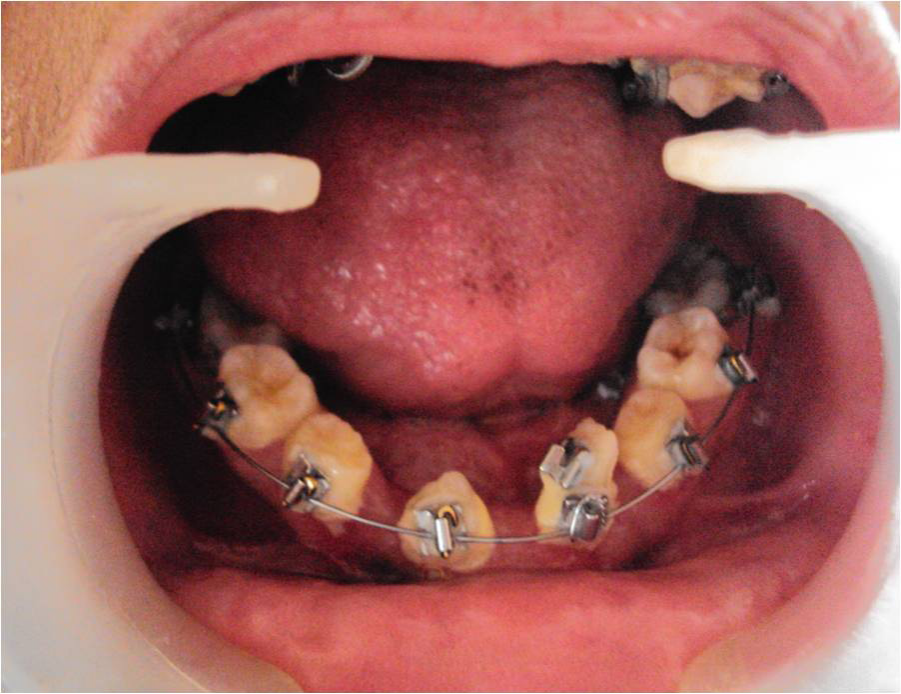
**Six month recall - note the erupted permanent teeth assisted by orthodontic brackets**.

After six months, the permanent teeth were erupting assisted by the orthodontic brackets and arch wire (Figure 8). Despite thorough oral hygiene instructions and maintenance during every follow-up visit, our patient suffered from poor oral hygiene due to the bonded orthodontic brackets and wires.

Under an aggressive oral hygiene maintenance program, our patient is followed-up periodically for further treatment.

## Discussion

Cleidocranial dysplasia is a well defined clinical phenotype arising from deregulation of intramembranous and endochondral ossification due to a mutation in Cbfa1. Hypermobility of the shoulders, abnormal clavicles, wormian bones and supernumerary teeth are seen to be consistent features of cleidocranial dysplasia. In our case, the pathognomonic features, like the absence of clavicles, broad skull sutures and numerous impacted and supernumerary teeth, were present [2,5].

It has been suggested that 70% of patients with cleidocranial dysplasia have a point mutation involving *Runx2 *and 13% have a deletion. In patients whose mutations are not found by traditional sequencing, the deletion/duplication assay, either Reverse transcription - quantitative real time polymerase chain reaction (RT-qPCR) or Multiplex Ligation-dependent Probe Amplification (MLPA), needs to be done [10-12]. Though it has been reported that individuals with central core disease could have cytogenetically visible complex chromosome rearrangements [13], in the present case the chromosomal analysis and gene mapping were normal. In order to identify the mutations in the *Runx2*, molecular genetic analysis is recommended.

Dental findings in cleidocranial dysplasia are characterized by a decreased eruptive force of both primary and permanent dentition, prolonged retention of primary teeth [3] and an increase in odontogenesis leading to an excessive number of supernumerary teeth [14]. It has been proposed that the supernumerary teeth should be diagnosed and removed as early as possible because they will impede the normal eruption of permanent teeth [15]. This suggestion was followed in our case. Further, an anomaly in the eruption of the anterior teeth may interfere with facial aesthetics and lead to other clinical problems.

The treatment objective in our case was to consider both the physical and psychological aspects of our patient. Redistribution of the space in her oral cavity and guidance of the permanent teeth to erupt in a proper alignment were planned. These were achieved by extracting the retained teeth and surgical exposure followed by orthodontic traction for the eruption of the permanent teeth [16]. Simultaneously, the psychological well-being of our patient was taken care of through behavior management methods and counseling, ultimately resulting in an improvement in her self-image and confidence.

Stabilization of the periodontal health of her permanent teeth and necessary orthodontic treatment will be continued in further follow-up appointments [17].

## Conclusion

Cleidocranial dysplasia patients with compromised aesthetics are usually seen as an unexpected event in the course of observing or treating a patient. Early diagnosis allows proper orientation to the treatment and offers a better life quality. A holistic approach takes care of all the aspects, including the primary pathology and the psychological aspects.

### Consent

Written informed consent was obtained from our patient's parents for publication of this case report and any accompanying images. A copy of the written consent is available for review by the Editor-in-Chief of this journal.

## Competing interests

The authors declare that they have no competing interests.

## Authors' contributions

NC analyzed and interpreted the patient's data regarding the associated dental problems. BSS, SM, NHK and RY performed jointly the treatment procedures and were major contributors in writing the manuscript. All authors read and approved the final manuscript.
